# Hsa_circ_0011385 knockdown represses cell proliferation in hepatocellular carcinoma

**DOI:** 10.1038/s41420-021-00664-0

**Published:** 2021-10-01

**Authors:** Chuangye Ni, Shikun Yang, Yang Ji, Yunfei Duan, Wenjie Yang, Xinchen Yang, Min Li, Jun Xie, Chuanyong Zhang, Yunjie Lu, Hao Lu

**Affiliations:** 1 Hepatobiliary/Liver Transplantation Center, The First Affiliated Hospital of Nanjing Medical University; Key Laboratory of Liver Transplantation, Chinese Academy of Medical Sciences, Nanjing, 210029 China; 2 Department of Hepatobiliary Surgery, The First People’s Hospital of Changzhou, The Third Hospital Affiliated to Soochow University, Changzhou, 213000 China

**Keywords:** Hepatocellular carcinoma, Hepatocellular carcinoma

## Abstract

Circular RNAs (circRNAs), continuous loops of single-stranded RNA, regulate gene expression during the development of various cancers. However, the function of circRNAs in hepatocellular carcinoma (HCC) is rarely discussed. Quantitative real-time polymerase chain reaction (qRT-PCR) was used to determine the mRNA levels of circ_0011385, miR-361-3p, and STC2 in 96 pairs of HCC tissues (tumor tissues and adjacent normal tissues), HCC cell lines, and L02 (human normal liver cell line) cells. The relationships between circ_0011385 expression and clinical features of HCC were evaluated. Functional experiments in vitro or in vivo were used to evaluate the biological function of circ_0011385. Bioinformatics analysis was performed to predict miRNAs and mRNAs sponged by circ_0011385. RNA immunoprecipitation (RIP) and dual-luciferase reporter gene assays were used to elucidate the interactions among circ_0011385, miR-361-3p, and STC2 (stanniocalcin 2). ChIP and dual-luciferase reporter gene assays were used to identify the upstream regulator of circ_0011385. High expression of circ_0011385 was observed in HCC tissues and cell lines and was significantly associated with tumor size, TNM stage, and prognosis. In addition, inhibition of circ_0011385 expression prevented the proliferation of HCC cells in vitro and in vivo. Circ_0011385 sponged miR-361-3p, thereby regulating the mRNA expression of STC2. In addition, the transcription of circ_0011385 was regulated by SP3. Circ_0011385 knockdown suppressed cell proliferation and tumor activity in HCC. Circ_0011385 may therefore serve as a new biomarker in the diagnosis and treatment of HCC.

## Introduction

Hepatocellular carcinoma (HCC) is the third most common cancer in men worldwide [1, 2]. The incidence of HCC ranks third among all cancers in China, and HCC is responsible for more than 50% of new cases and deaths worldwide [3]. HCC can be caused by HBV infection and cirrhosis [4]. In the past few decades, substantial efforts have attempted to improve the prognosis of HCC. Although many novel alternative therapies have been developed, surgical resection remains the main choice for HCC [5]. However, the 5-year survival rate remains very low (<20%) [6]. Therefore, a better understanding of its molecular mechanisms is needed to develop more effective treatments for HCC.

Circular RNAs (circRNAs) are non-coding RNA molecules mainly derived from the reverse splicing of pre-mRNA [7]. CircRNAs have been shown to be dysregulated in the growth of tumors, including HCC [8, 9]. For instance, circCAMSAP1 promotes HCC progression, and the circ_0008305/miR-660/BAG5 axis functions in HCC tumorigenesis [10, 11]. However, the function and mechanism of circRNAs in HCC are not fully understood. Circ_0011385 originates from the eukaryotic translation initiation factor 3 subunit I (EIF3I) gene and is located on chr1:32691771–32692131. Circ_0011385 facilitates proliferation, invasion, and migration while inhibiting the arrest and apoptosis of thyroid cancer cells [12]. However, the role of circ_0011385 in HCC has not been elucidated.

MicroRNAs (miRNAs) are short, mostly noncoding RNAs approximately 22 nucleotides in length [13]. Dysregulated miRNAs can have oncogenic or suppressive effects on tumors. Studies increasingly indicate that circRNAs rely on competitive endogenous RNAs (ceRNAs) to modulate the expression of downstream miRNAs [14]. In HCC, circPVT1 regulates cellular proliferation, apoptosis, and glycolysis via the miR-377/TRIM23 axis, and circLDLR knockdown suppresses HCC progression through the miR-7/RNF38 axis [15, 16]. Moreover, miRNAs have been shown to regulate mRNA levels by targeting their 3′-untranslated regions (UTRs) [17].

On the basis of the GSE97332 dataset, circ_0011385 was identified as a dysregulated circRNA in HCC and further verified at the tissue and cellular levels. Circ_0011385 accelerated cell proliferation in vivo and in vitro, and it sponges miR-361-3p, thereby regulating the expression of STC2. In addition, SP3 might serve as a transcriptional activator of circ_0011385.

## Results

### Circ_0011385 is upregulated in HCC tissues and cells

**Fig. 1:**
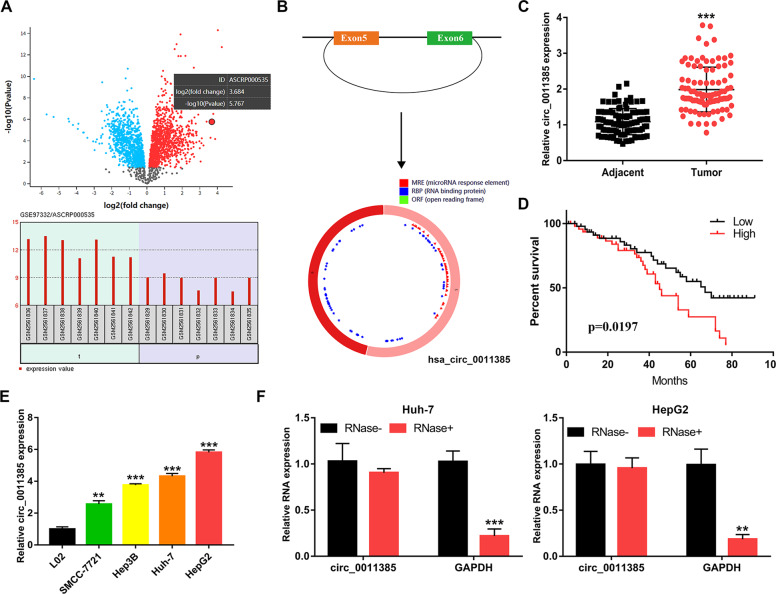
Circ_0011385 is upregulated in HCC tissues and cells. **A** Dysregulated circRNAs in GSE97332 were assessed, and circ_0011385 was chosen for study. **B** Schematic drawing illustrating that hsa_circ_0011385 arises from exons 5 and 6 of the EIF3I gene. **C** Validated circ_0011385 expression in HCC tissues and adjacent tissues. **D** Overall survival analysis based on circ_0011385 expression in 96 patients with HCC. **E** Relative circ_0011385 expression in HCC and L02 cell lines. **F** The expression levels of circ_0011385 in Huh-7 and HepG2 cell lines after treatment with RNase R. Data (n = 5) are the means ± SD. *P < 0.05, **P < 0.01, ***P < 0.001.

The abnormally expressed circRNAs in HCC tissues were screened from GSE97332 data. On the basis of the |logFC| and adjusted *P* value, we found that circ_0011385 expression was significantly higher in tumor tissues (Fig. 1A). According to the UCSC Genome Browser, circ_0011385 was generated from back-splicing of the 5th and 6th exons of the EIF3I gene (Fig. 1B). We then performed qRT-PCR analysis on 96 patients with HCC, which indicated that circ_0011385 expression was significantly elevated in HCC tissues (Fig. 1C). To demonstrate the clinical significance of the upregulation of circ_0011385, we analyzed the clinicopathological characteristics of patients with HCC. Using the median expression level as a cut-off value, we divided our patient cohort into two groups: a high expression group (*n* = 54) and a low expression group (*n* = 42). As shown in Table 1, the expression level of circ_0011385 varied with tumor size and TNM stage, but not with age, sex, HBV infection, and liver cirrhosis. In addition, patients with high expression of circ_0011385 had a poorer prognosis than those with low expression (Fig. 1D). Moreover, circ_0011385 was upregulated in all four HCC cell lines (SMCC-7721, Hep3B, Huh-7, and HepG2) (Fig. 1E). After RNase R treatment, the expression levels of circ_0011385 in Huh-7 and HepG2 cell lines did not change significantly, thus, indicating that circ_0011385 exists stably in cells (Fig. 1F). The above results suggested that circ_0011385 may be involved in the pathogenesis of HCC.

**Table 1:** The correlation between hsa_circ_0011385 expression and clinicopathological features in 96 HCC patients.

	All cases	has_circ_0011385 expression		
Features	Total	Low	High	*P* value
Total number	96	42	54	
*Age*				
>55	58	28	30	0.269
≦55	38	14	24	
*Gender*				
Male	50	24	26	0.381
Female	46	18	28	
*HBV infection*				
Absent	41	22	19	0.091
Present	55	20	35	
*Liver cirrhosis*				
With	54	25	29	0.568
Without	42	17	25	
*Tumor size (cm)*				
>5	44	14	30	0.001
≦5	42	28	14	
TNM stage				
I + II	40	24	16	0.007
III + IV	56	18	38	

### Knockdown of circ_0011385 hinders the proliferation of HCC cells

**Fig. 2:**
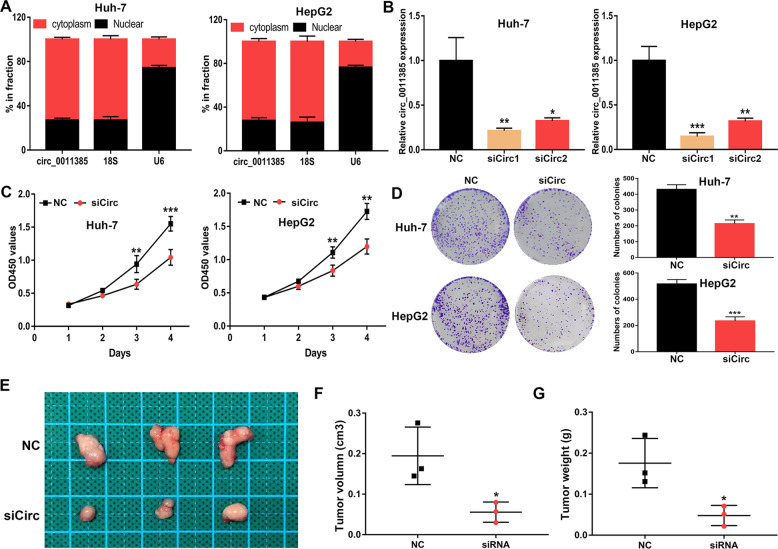
Knockdown of circ_0011385 hinders the proliferation of HCC cells. **A** Nuclear and cytoplasmic mRNA fraction experiment, displaying the location of circ_0011385 in Huh-7 and HepG2 cells. **B** The expression levels of circ_0011385 in transfected Huh-7 and HepG2 cells. **C**, **D** CCK-8 and colony formation assays evaluating cell proliferative effects of circ_0011385 in Huh-7 and HepG2 cells. **E**–**G** Image of xenograft tumors. Tumor volume and tumor weight were measured in the control and circ-0011385 groups (*n* = 3/group). Data (*n* = 5) are the means ± SD. *P < 0.05, **P < 0.01, ***P < 0.001.

Because Huh-7 and HepG2 cells demonstrated the highest expression of circ_0011385, we selected these cell lines for further in vitro and in vivo functional analyses. QRT-PCR indicated that circ_0011385 was distributed mainly in the cytoplasm (Fig. 2A). The Huh-7 and HepG2 cell lines were transfected with NC, siCirc1, and siCirc2. Both siCirc1 and siCirc2 decreased the expression of circ_0011385 in the cells, and we chose siCirc2 for further study, given its highest knockdown efficiency (Fig. 2B). Knockdown of circ_0011385 significantly inhibited cell proliferation (Fig. 2C). In addition, low expression of circ_0011385 significantly inhibited the colony-forming ability of Huh-7 and HepG2 cells (Fig. 2D). In vivo, the tumor-forming ability of the siRNA group was significantly weaker than that of the NC group (Fig. 2E). The volume and weight of the tumors in the siRNA group were significantly lower than those in the NC group (Fig. 2F, G). These data demonstrated that the proliferative ability of HCC cells significantly decreased after circ_0011385 was knocked down.

### MiR-361-3p is sponged by circ_0011385

**Fig. 3:**
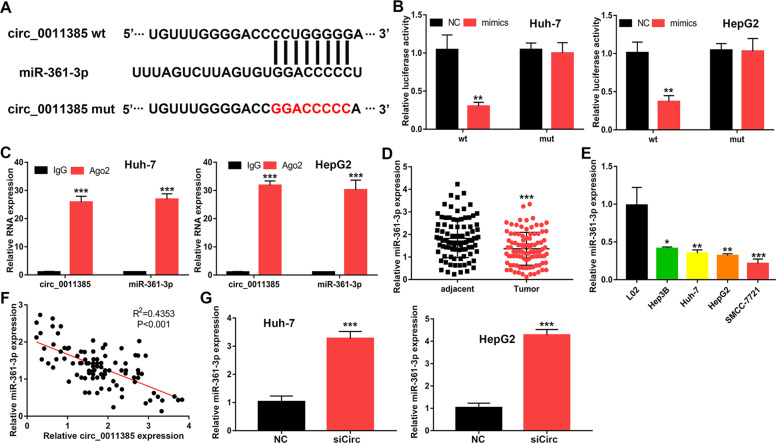
miR‐361-3p is the target of circ_0011385. **A** Bioinformatics tools indicated the complementary binding sites of miR‐361-3p and circ_0011385. **B** Luciferase reporter assays showing the targeting of miR‐361-3p with circ_0011385 in Huh-7 and HepG2 cells. **C** RIP and qRT-PCR assays measuring the expression differences in circ_0011385 and miR-361-3p in Huh-7 and HepG2 cells. **D** Relative miR-361-3p expression in HCC tissues and adjacent tissues. **E** Relative miR-361-3p expression in HCC cells and L02. **F** Correlation between miR‐361-3p and circ_0011385, calculated with Spearman’s rank correlation coefficients. **G** qRT‐PCR of miR-361-3p expression after circ_0011385 knockdown in Huh-7 and HepG2 cells. Data (*n* = 5) are the means ± SD. *P < 0.05, **P < 0.01, ***P < 0.001.

We conducted further experiments to clarify how circ_0011385 exerts its effect. Starbase predicted that miR-361-3p might be a potential target of circ_0011385 (Fig. 3A). To further verify the interaction between circ_0011385 and miR-361-3p, we performed luciferase reporter gene assays. The overexpression of miR-361-3p in HCC cells significantly repressed the luciferase activity in the wild-type (WT) group, but not the mutant (MUT) group (Fig. 3B). RIP assays suggested that circ_0011385 and miR-361-3p were more enriched by Ago2 immunoprecipitation than IgG immunoprecipitation (Fig. 3C). In addition, the expression levels of miR-361-3p in HCC tissues and cells were significantly elevated and were negatively correlated with that of circ_0011385 and miR-361-3p (Fig. 3D–F). Moreover, the knockdown of circ_0011385 significantly increased the expression of miR-361-3p in Huh-7 and HepG2 cells (Fig. 3G). These results indicated that circ_0011385 regulates miR-361-3p expression through direct sponging.

### MiR-361-3p inhibits the proliferation of HCC cells

**Fig. 4:**
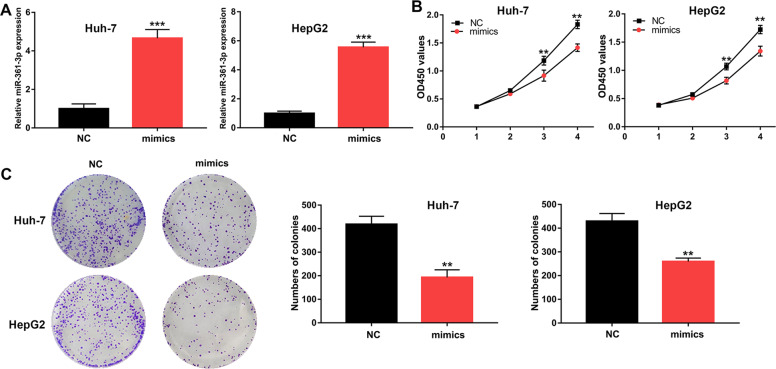
miR-361-3p inhibits cell proliferation in HCC cells. **A** Relative miR-361-3p expression in transfected Huh-7 and HepG2 cells. **B**, **C** CCK-8 and colony formation assays evaluating the cell proliferative effects of miR-361-3p in Huh-7 and HepG2 cells. Data (*n* = 5) are the means ± SD. *P < 0.05, **P < 0.01, ***P < 0.001.

To explore how miR-361-3p regulates the proliferation of HCC cells, we transfected miR-361-3p mimics into Huh-7 and HepG2 cells, and verified the transfection efficiency with qRT-PCR (Fig. 4A). CCK-8 and colony formation assays demonstrated that after overexpression of miR-361-3p, the proliferative ability of HCC cells significantly decreased (Fig. 4B, C).

### STC2 is a downstream target of miR-361-3p

**Fig. 5:**
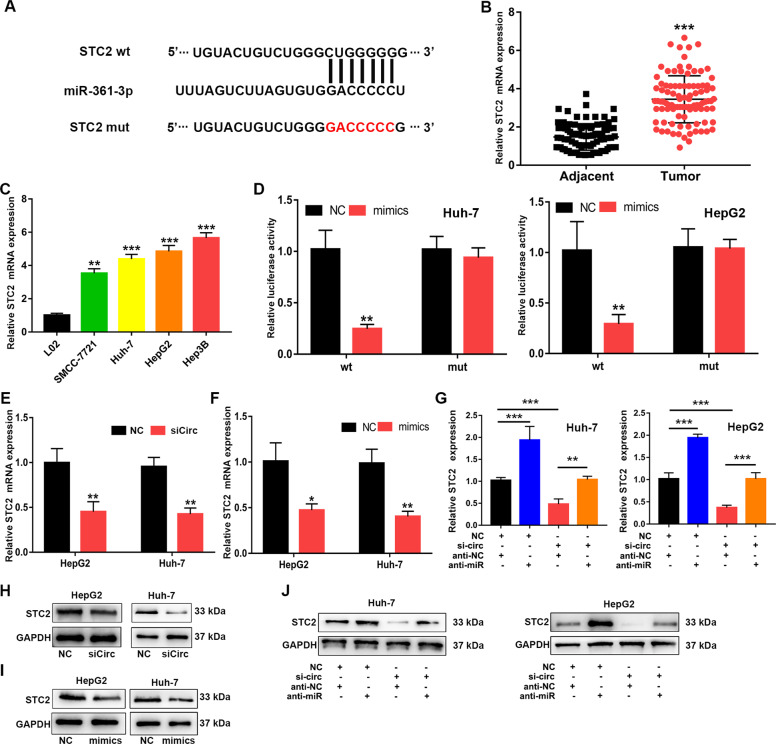
STC2 is the target of miR‐361-3p. **A** Bioinformatics tools indicated the complementary binding sites of miR‐361-3p with STC2. **B**, **C** Relative STC2 expression in HCC tissues and cells. **D** Luciferase reporter assays showing the targeting of miR‐361-3p with STC2 in Huh-7 and HepG2 cells. **E**–**G** qRT‐PCR showing STC2 mRNA expression in transfected Huh-7 and HepG2 cells. **H**–**J** Western blotting showing STC2 protein expression in transfected Huh-7 and HepG2 cells. Data (*n* = 5) are the means ± SD. *P < 0.05, **P < 0.01, ***P < 0.001.

To further study the regulatory role of miR-361-3p in HCC cells, we used Starbase to predict the potential targets of miR-361-3p (Fig. 5A). STC2 mRNA was highly expressed in HCC tissues and cell lines (Fig. 5B, C). In addition, WT or MUT STC2–3′ UTR was inserted into a pMIR-REPORT luciferase vector, which in turn was introduced into Huh-7 and HepG2 cells with expressed miR-361-3p mimics or NC. As shown in Fig. 5D, miR-361-3p mimics significantly decreased the fluorescence intensity in the WT group but did not change that in the MUT group. After circ_0011385 knockdown, the expression level of STC2 decreased (Fig. 5E, H). Similarly, after overexpression of miR-361-3p, the mRNA and protein levels of STC2 decreased significantly (Fig. 5F, I). In contrast, the decrease in STC2 caused by circ_0011385 inhibition was abolished by miR-361-3p knockdown (Fig. 5G, J). Together, our results indicated that the expression of STC2 is regulated by miR-361-3p and circ_0011385.

### SP3 is a transcriptional regulator of circ_0011385

**Fig. 6:**
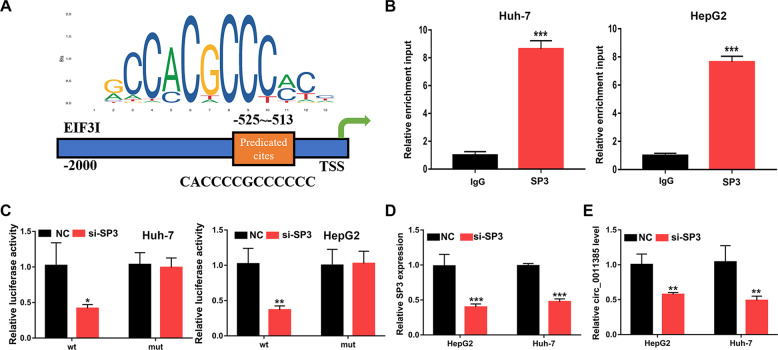
SP3 activates circ_0011385 expression. **A** A potential binding site for SP3 in the circ_0011385 promoter. **B** ChIP assay indicating SP3 occupancy of the circ_0011385 promoter. **C** Relative luciferase activity analyzed in Huh-7 and HepG2 cells. **D**, **E** Relative STC2 and circ_0011385 expression in Huh-7 and HepG2 cells. Data (*n* = 5) are the means ± SD. *P < 0.05, **P < 0.01, ***P < 0.001.

To describe the transcriptional regulation of circ_0011385 in HCC, we searched the JASPAR database for potential transcription factors (Fig. 6A). SP3 was selected for subsequent analysis. According to ChIP analysis, the promoter region of circ_0011385 was pulled down by the SP3 specific antibody, but not the control antibody (Fig. 6B). According to dual-luciferase reporter gene assays, si-SP3 significantly weakened the fluorescence in the WT group (Fig. 6C). In the HCC cell line, the expression of SP3 and circ_0011385 decreased after transfection of si-SP3 (Fig. 6D, E). These findings implied that SP3 is a transcriptional activator of circ_0011385.

## Discussion

Emerging evidence indicates that circRNA is abnormally expressed in the development of HCC [18, 19]. On the basis of the functions of circRNA in HCC, many circRNAs have been discovered as biomarkers in recent years [20]. In this study, we identified circRNA hsa_circ_0011385 from the GSE97332 dataset. We first found that circ_0011385 was significantly upregulated in HCC tissues and cell lines. Circ_0011385 was found to regulate cell proliferation in vitro and inhibit tumor progression in vivo. These findings indicated that circ_0039053 is oncogenic in the development of HCC.

CircRNAs target miRNAs to function. Here, we revealed that circ_0011385 can be used as a ceRNA against miR-361-3p, thus, increasing the expression of STC2 mRNA in HCC. Studies have shown that miR-361-3p suppresses the progression of some human tumors, including multiple myeloma, lymphoma, and prostate cancer [21–24]. MiR-361-3p expression is upregulated in HCC cells and liver tumor-initiating cells. The dysregulated expression of miR-361-3p enhances the self-renewal and tumorigenic liver tumor-initiating cells [25, 26]. In agreement with findings from previous studies, we also observed a decrease in miR-361-3p expression in HCC tissues and cell lines. Meanwhile, miR-361-3p overexpression inhibited the proliferation of HCC cells. These findings indicate that circ_0011385 may serve as a miR-361-3p sponge that drives the progression of HCC.

STC2, a mammalian stanniocalcin, is located on chromosome 5q35.1 and is involved in a variety of biological processes, including calcium regulation, cell proliferation and apoptosis, inflammation, endoplasmic reticulum/oxidative stress, metabolism, and cancer processes [27–30]. The relative expression level of STC2 in hepatocellular carcinoma tissue is associated with tumor size, hepatocellular carcinoma stage, metastasis, and differentiation. The median survival times of patients with HCC with high STC2 expression is also relatively shorter [31]. In addition, ectopic expression of STC2 promotes proliferation and colony formation of HCC cells. The expression of STC2 also regulates the switch from G1 to S and the protein levels of cyclin D1 and pERK1/2, thus, indicating that STC2 has a direct role in the progression and metastasis of hepatocellular carcinoma [32]. In this study, STC2 expression rose in HCC tissues and cell lines, again verifying the results in previous studies. Moreover, the mRNA level of STC2 was regulated by circ_0011385 and miR-361-3p.

SP3 is a transcription factor whose protein expression is usually higher in cancer cells than in normal cells. SP3 can also enhance or repress the activity of promoters of genes involved in differentiation, the cell cycle, and oncogenesis [33]. SP3 protein contains a zinc finger DNA binding domain and several transactivation domains, with four transcript variants encoding different isoforms [34]. SP3 binds GT- and GC-box promoter elements and competes with SP1 for binding the GC-box promoter—a mechanism that regulates gene transcription [35]. SP3 is dysregulated in various cancers and is highly expressed in HCC [36–39]. Sp3 decreases the sensitivity of Hep3B cells to chemotherapy, and it uses MALAT1 to regulate HCC [39, 40]. In this study, we report the first evidence that SP3 increases the expression of circ_0011385, thus, affecting the expression of miR-361-3p and STC2 mRNA.

In summary, circ_0011385 is an oncogenic molecule that, after activation by SP3, regulates the proliferation and invasion of HCC cells through the miR-361-3p/STC2 axis. Our findings provide potential new biomarkers for the treatment of HCC.

## Materials and methods

### HCC tissue sampling

The study was approved by the Medical Ethics Committee of the First Affiliated Hospital of Nanjing Medical University, and written informed consent was obtained from all patients. Tissue samples of 96 patients who had undergone surgery between January 2018 and November 2019 were collected. Chemotherapy or radiotherapy had not been performed in these patients preoperatively. Tissues were sampled and kept in liquid nitrogen until use. The clinicopathological data and survival information of all patients were also collected.

### Cell culture

HCC cell lines (SMCC-7721, Huh-7, HepG2, and Hep3B) and one normal liver cell line (L02) were provided by the Shanghai Academy of Sciences (Shanghai, China). All cells were grown at 37 °C in RPMI 1640 medium (Gibco, NY, USA) mixed with 10% fetal bovine serum (Gibco), 100 U/mL penicillin (Gibco), and 100 mg/mL streptomycin (Gibco), and were incubated in a 5% CO_2_ atmosphere.

### Total RNA extraction and reverse-transcription quantitative real-time polymerase chain reaction (qRT-PCR)

Total RNA was extracted from tissues and cells with Trizol reagent. After RNA was reverse transcribed into single-stranded cDNA, qRT-PCR was performed with SYBR Green Master Mix (Takara, Tokyo, Japan). GAPDH was used as an internal reference for circRNA and mRNA, and U6 was used as an internal reference for mRNA. The relative expression level was calculated with the 2^−∆∆CT^ method. The primers used are listed in Table S1.

### Western blot analysis

Cellular protein was extracted with RIPA lysis buffer according to the manufacturer’s protocol (Beyotime, Shanghai, China). The extracted protein was separated with 10% sodium dodecyl sulfate-polyacrylamide gel electrophoresis and transferred onto a polyvinylidene fluoride membrane (Bio-Rad, CA, USA). Rabbit anti-STC2 polyclonal antibody was used, and anti-GAPDH was used as an internal control (Proteintech Group, Chicago, IL, USA). Protein expression levels were detected with HRP conjugated secondary antibodies and ECL Plus (EMD Millipore, Billerica, MA, USA).

### RNase R treatment assays

RNA and RNase R (Geneseed Biotech, Guangzhou, China) were mixed and incubated for 20 min at 37 °C. QRT-PCR was performed again to measure the mRNA levels of circ_0011385 and GAPDH expression.

### Subcellular localization

A PARIS Kit (Invitrogen, Carlsbad, CA, USA) was used to define the subcellular localization of circ_0011385.

### Cell transfection

Three siRNAs (siCirc1, siCirc2, and si-SP3) and negative control siRNA (NC) (RiboBio, Guangzhou, China) were transfected into Huh-7 and HepG2 cells. After 48 h of transfection, qRT-PCR was performed to assess the transfection efficiency. The cells were transfected with Si-SP3, NC, miR-361-3p mimics, and NC provided by the same company, with Lipofectamine 3000 (Invitrogen) as the transfection reagent. The sequences used for transfection in the article were listed in Table S2.

### CCK-8 assays

After 48 h of transfection, cellular proliferation was analyzed with a Cell Counting Kit-8 (CCK-8, Vazyme, Nanjing, China). In brief, the transfected cells were seeded into a 96-well plate (Invitrogen) (1 × 10^3^ per well), and subsequently measured at 450 nm OD at 24, 48, 72, and 96 h. After 10 μl of CCK-8 reagent was injected into each well, the cells were incubated for another hour at 37 °C.

### Colony formation assays

A six-well plate (Invitrogen) was used to evaluate colony formation ability. After resuspension, the transfected cells were seeded on 6-well plates (500 cells/well). After culturing under a constant temperature for 10 days, the cells were washed with PBS three times, fixed with methanol for 10 min, stained with 0.1% crystal violet, photographed, and counted.

### Xenograft tumor model

Six male nude mice were prepared in the Animal Experiment Center of Nanjing Medical University. The transfected HCC cells (1 million cells/mouse) were inoculated into the left armpit of each nude mouse. All nude mice were sacrificed 24 days later, the transplanted tumors were removed and imaged, and tumor weight and volume were measured.

### Bioinformatics analysis

The GSE97332 dataset, containing the expression data for circRNAs in HCC tissues and normal tissues, was downloaded from the GEO database and analyzed online with GEO2R. | logFC | <1 and adjusted *P* value < 0.05 were used as the screening criteria to search for dysregulated circRNAs. Starbase (http://starbase.sysu.edu.cn/) was used to predict the target genes of circ_0011385 and miR-361-3p. JASPAR (http://jaspar.genereg.net/) was selected to search for possible upstream regulators.

### Dual-luciferase reporter gene assays

We used the online bioinformatics website Starbase to predict the binding sites. Recombinant luciferase vectors carrying WT and MUT circ_0011385 and STC2 sequences were constructed. Then the WT and MUT were inserted into the pMIR-REPORT vector (Ambion, Waltham, MA, USA). Huh-7 and HepG2 cells were co-transfected with si-SP3, miR-361-3p mimics, or NC with Lipofectamine 3000 (Invitrogen). After 48 hours of incubation, the dual-luciferase reporter gene detection system was used to detect luciferase activity (Promega Corporation).

### RNA immunoprecipitation (RIP) analysis

RIP analysis was performed with a RIP kit (Merck, Darmstadt, Germany). In brief, Huh-7 and cells were lysed with RIP lysis buffer, and the lysates were incubated with magnetic beads pre-conjugated with anti-AGO2 or anti-IgG antibody at 4 °C for 6 h. Then, the magnetic beads were washed and digested with proteinase K. QRT-PCR was conducted for analysis of purified RNA.

### Chromatin immunoprecipitation (ChIP)

ChIP was performed with a ChIP Chromatin Immunoprecipitation Kit (Millipore, Massachusetts, USA). Chromatin was cross-linked with formaldehyde, sonicated into small fragments, and immunoprecipitated with anti-SP3 or anti-IgG antibodies bound to magnetic beads. The enriched fragments were collected by qRT-PCR after decrosslinking.

### Statistical analysis

All quantitative data from at least three independent experiments were expressed as the mean ± standard deviation (SD). Statistical analysis was performed in GraphPad Prism 8.0 software (GraphPad Software, La Jolla, CA, USA) and SPSS 25.0 (SPSS, Chicago, IL, USA). One-way analysis of variance and Student’s *t* test were used to assess for between-group differences, chi-square test was used for the association between circRNA expression and clinicopathological parameters of HCC, and Spearman’s rank correlation coefficient was used for correlation analysis. *P* < 0.05 was considered statistically significant.

## Supplementary information

Table S1

Table S2

Dataset 1

Dataset 2

Dataset 3

Dataset 4

## Data Availability

The data that support the findings of this study are available on request from the corresponding author. The data are not publicly available due to privacy or ethical restrictions.
