# Supplementary material for: Hsa_circ_0011385 knockdown represses cell proliferation in hepatocellular carcinoma
Source: Cell Death Discov. 2021 Oct 1;7:270. doi: 10.1038/s41420-021-00664-0 (PMC8486831; doi:10.1038/s41420-021-00664-0)
Supplement: Supplementary file 1 — Table S1 [file 41420_2021_664_MOESM1_ESM.docx]

**Table S1. Primers used for RT-qPCR in the present study**

| Genes | Forward primer | Reverse primer |
| --- | --- | --- |
| Hsa_circ_0011385 | TGACAACAATGAGCCCTACA | TTTCCTTGGCACTATACTGG |
| miR-361-3p | ACACTCCAGCTGGGTCCCCCAGGTGTGATTCTG | CTCAACTGGTGTCGTGGAGTCGGCAATTCAGTTGAGAAATCAGA |
| STC2 | CGGAAGTGTCCAGCCATCAAGG | GCAGCAGTCACACACAGTCA |
| SP3 | CTTACTTGCCTCTGGAACACCT | ACCAAGAGGCACATTAGCAAC |
| β-actin | TGACGTGGACATCCGCAAAG | CTGGAAGGTGGACAGCGAGG |
