# Supplementary material for: Hsa_circ_0011385 knockdown represses cell proliferation in hepatocellular carcinoma
Source: Cell Death Discov. 2021 Oct 1;7:270. doi: 10.1038/s41420-021-00664-0 (PMC8486831; doi:10.1038/s41420-021-00664-0)
Supplement: Supplementary file 2 — Table S2 [file 41420_2021_664_MOESM2_ESM.docx]

**Table S2. Sequences used for cell transfection**

| Target gene | Sequences |
| --- | --- |
| si-circ1 | UGAGUAAGACCGGAAAAGGUU |
| si-circ2 | UACUUAAUCUGCGUAAUGGAC |
| miR-361-3p mimics | UCCCCCAGGUGUGAUUCUGAUUU |
| anti-miR-361-3p | AAAUCAGAAUCACACCUGGGGGA |
| si-SP3 | CAGUCAAUCAAAAUAGGAAAA |
