# Supplementary material for: Hsa_circ_0011385 knockdown represses cell proliferation in hepatocellular carcinoma
Source: Cell Death Discov. 2021 Oct 1;7:270. doi: 10.1038/s41420-021-00664-0 (PMC8486831; doi:10.1038/s41420-021-00664-0)
Supplement: Supplementary file 6 — Dataset 4 [file 41420_2021_664_MOESM6_ESM.pdf]

# International Science Editing

***www.internationalscienceediting.com***

**DATE:** August 14, 2021

Compuscript Ltd
T/A International Science Editing
Bay K, Shannon Industrial Park West
Shannon, Co Clare
Ireland
Phone +353 61 472818    Fax +353 61 472688

To whom it may concern,

The paper “Hsa_circ_0011385 knockdown represses cell proliferation in hepatocellular carcinoma” Shikun Yang was edited by International Science Editing. We were asked not to edit the references. Please contact us if you would like to view the edited paper.

Kindest regards,

David Cushley.
